# Supplementary material for: Immunophenotypic but Not Genetic Changes Reclassify the Majority of Relapsed/Refractory Pediatric Cases of Early T-Cell Precursor Acute Lymphoblastic Leukemia
Source: Int J Mol Sci. 2024 May 21;25(11):5610. doi: 10.3390/ijms25115610 (PMC11171474; doi:10.3390/ijms25115610)
Supplement: Supplementary file 1 [file ijms-25-05610-s001.zip › ijms-2949966-supplementary.pdf]

Table S1. FISH probes used in the study

Rearrangements	Probes
BCL11B locus rearrangements	EmpireGenomics[1] BCL11B
KMT2A rearrangements	Kreatech[2] ON KMT2A<br>Kreatech ON KMT2A::MLLT4<br>Wuhan HealthCare Biotechnology[3] MLLT3::KMT2A<br>Cytocell[4] KMT2A::MLLT10
Aneuploidies	MetaSystems[5] XCE 4/10/17<br>MetaSystems XCE 7/8<br>Kreatech ON BCR::ABL<br>Cytocell ON TP53/CEN17
T-ALL-associated aberrations	Cytocell TLX3<br>Cytocell TCRalpha/delta<br>Cytocell TCRbeta<br>Cytocell TLX1<br>CytoTest[6] SIL-TAL<br>MetaSystems XL MYC
Miscellaneous aberrations	CytoTest ETV6<br>Cytocell RUNX1<br>Cytocell PICALM::MLLT10<br>EmpireGenomics HOXA@
Validation	Cytocell FOXO1

[1]Empire Genomics, Buffalo, NY, USA
[2]Leica Biosystems, Deer Park, IL, USA
[3]Wuhan HealthCare Biotechnology Co., Wuhan, Hubei, China
[4]Cytocell, Cambridge, UK
[5]MetaSystems Hard & Software GmbH, Altlussheim, Germany
[6]CytoTest Inc., Rockville, MD, USA

Table S2. Nucleotide sequences of primers and probes used in the study

Gene or gene fusion	Nucleotide sequence
ABL control	*ABL*-222F 5'-GGCCAGTGGAGATAACACTC-3'<br>*ABL*-351R 5'-GATGTAGTTGCTTGGGACCCA-3'<br>Probe *ABL* FAM-5'-CCATTTTTGGTTTGGGCTTCACACCATT-3'-BHQ1
TLX3 expression	*HOX11L2-F* 5'-AGACCTGGTTCCAAAACCG-3'<br>*HOX11L2-R* 5'-GCTGGATGGAGTCGTTGA-3'<br>Probe *HOX11L2-P* FAM-5'-CAGCTGCAACACGACGCCTTCCAA-3'-BHQ1
MNX1::ETV6	*MNX1*-604 5'-FATCAAGCTGGGCGCCGGCACCT-3'<br>*ETV6*-ex3R 5'-ATTTCAAACGTGTTGCTGTCAATTGG-3'
KMT2A::MLLT3	*KMT2A*-ex21 5'-GAGTCGAGAAGACAGTCCAGAGCT-3'<br>*MLLT3*-R2 5'-TGGCAGGACTGGGTTGTTC-3'

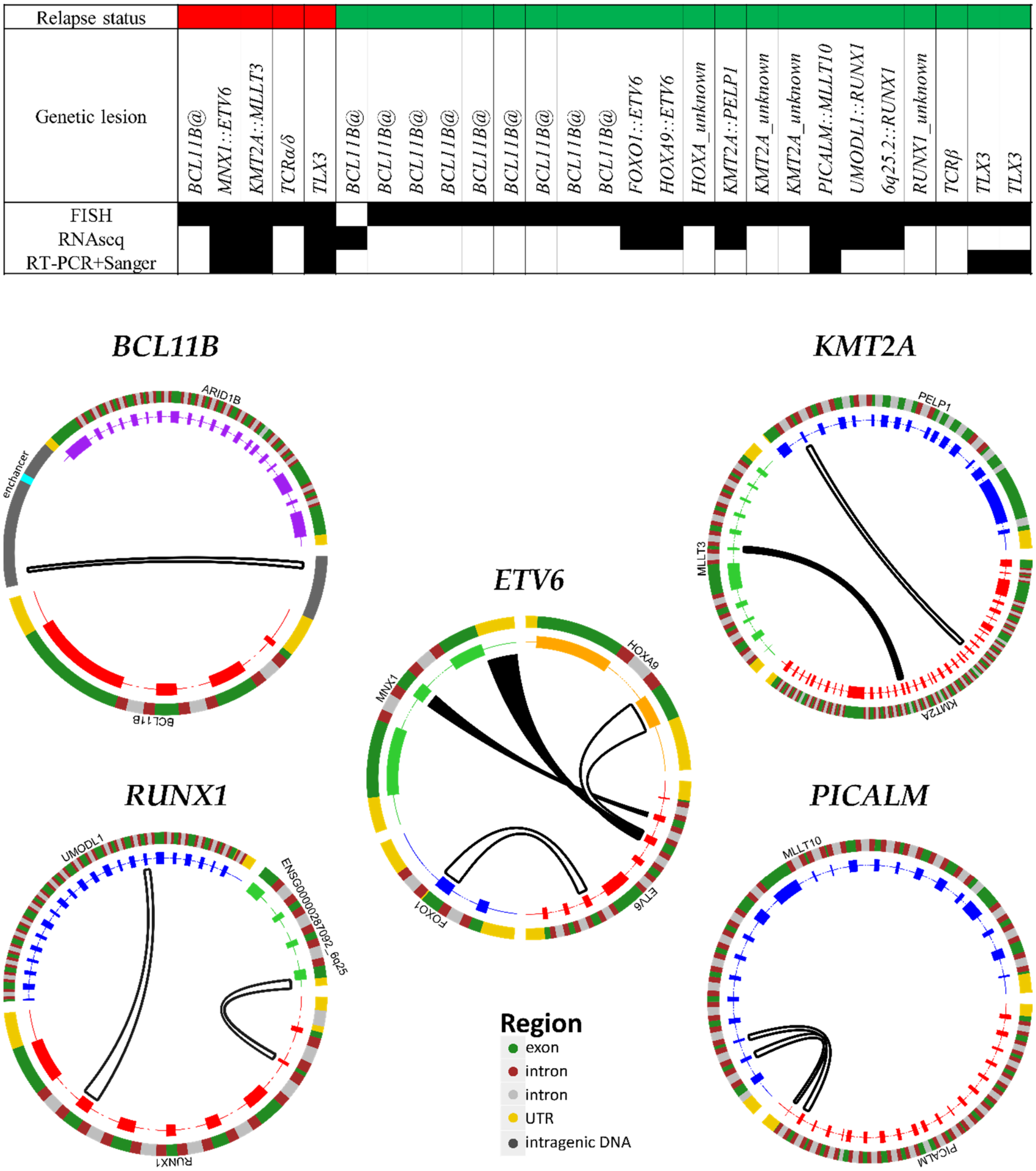

Figure S1. Recurrent genetic lesions at the initial examination in ETP-ALL with the respect to relapse status: detection methods and fusion structure (black for relapsed ETP-ALL cohort, white for non-relapsed ETP cohort).

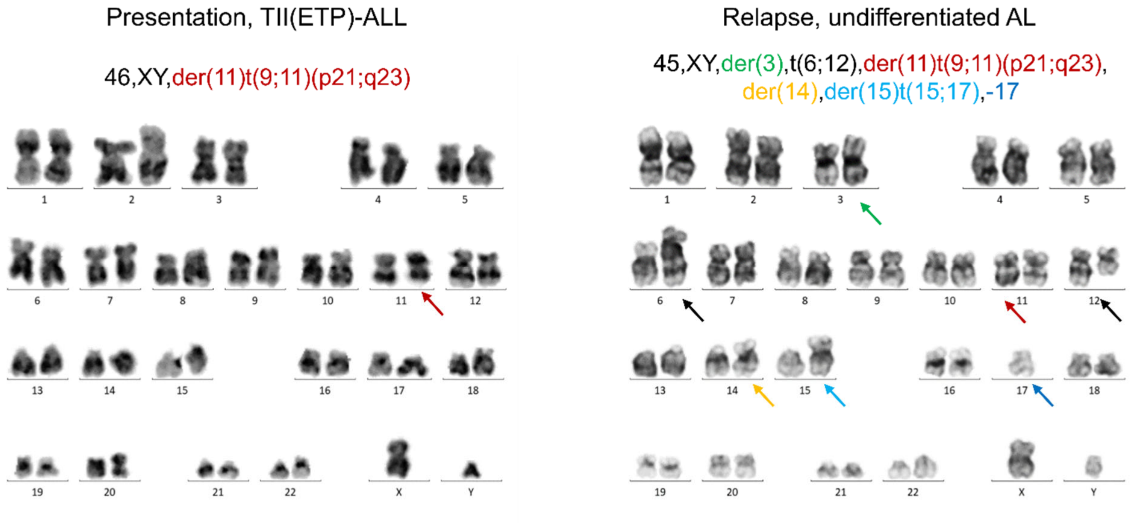

Figure S2. The accumulation of additional chromosomal aberrations in the *KMT2A::MLLT3*-positive ETP-ALL case relapse. The leading genetic event, namely t(9;11)(p21;q23), is marked in red at initial and relapse karyotyping. Additional chromosomal rearrangements emerged at relapse are marked in matching colors and arrows.

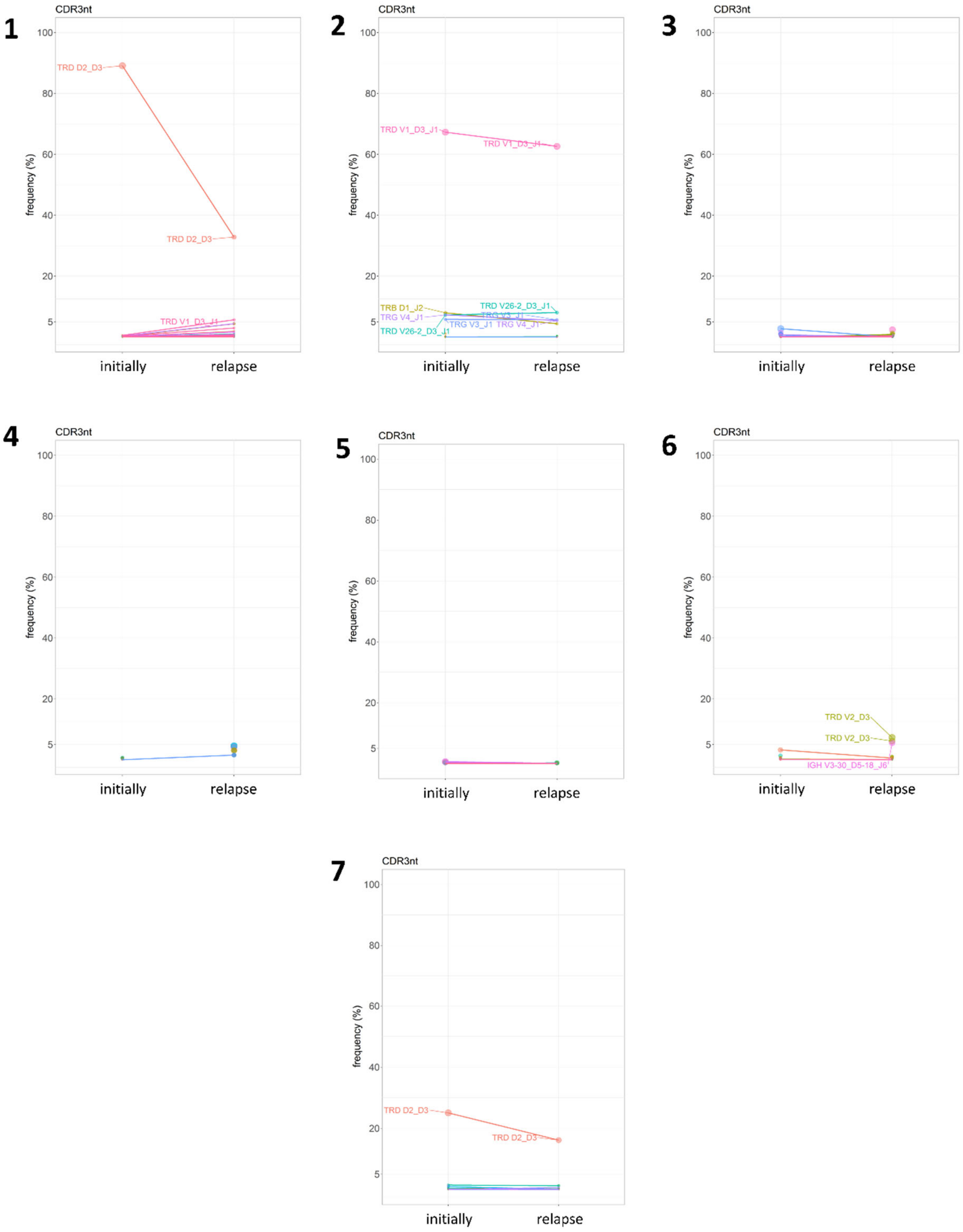

Figure S3. *TCR/BCR* clonal rearrangements in ETP-ALL at the initial examination and relapse: clonal dynamics shown for each studied patient, numbers 1-7 refer to patient's numbers in Table 4. Frequency is VAF among all identified rearrangements, circle size is VAF among identified rearrangements of particular fraction (immunoglobulins: *IGH*, *IGK*, *IGL*; *TCR*: *TRA*, *TRB*, *TRG*, *TRD*).
